# Editorial: Omics Solutions for Endocrine Disorders

**DOI:** 10.3389/fendo.2022.948991

**Published:** 2022-06-22

**Authors:** Stephen L. Atkin, Anna M. Halama

**Affiliations:** ^1^ Department of Research, Royal College of Surgeons in Ireland, Al Muharraq, Bahrain; ^2^ Department of Research, Weill Cornell Medicine- Qatar, Ar-Rayyan, Qatar

**Keywords:** omics, proteomic, transcriptomatics, systems biology, endocrinolgy, diabetes, genomics

Multifactorial processes contribute to the development of endocrinological disorders such as diabetes, polycystic ovary syndrome (PCOS) and some carcinomas. Frequently, standard clinical tests are insufficient for proper diagnosis and treatment. Therefore, strategies providing further insight into endocrinological disorders that support the clinical pipeline are required. Recently, omics technologies such as genomics, transcriptomics, proteomics, metabolomics and lipidomics have been introduced into the field of complex disorders to improve diagnostics and treatment (**Figure 1**). This special edition highlights the use of omic technologies in endocrinological disorders, including diabetes and its comorbidities, as well as obesity and PCOS, further emphasizing their potential to be implemented into the clinical pipeline. What this special edition also emphasizes is the differing methodology available for each of the “omic” approaches that may not be directly comparable, one example being in proteomics where some groups use aptamer-based technology whilst others may use mass spectroscopy, and therefore the need for standardization.

**Figure 1:**
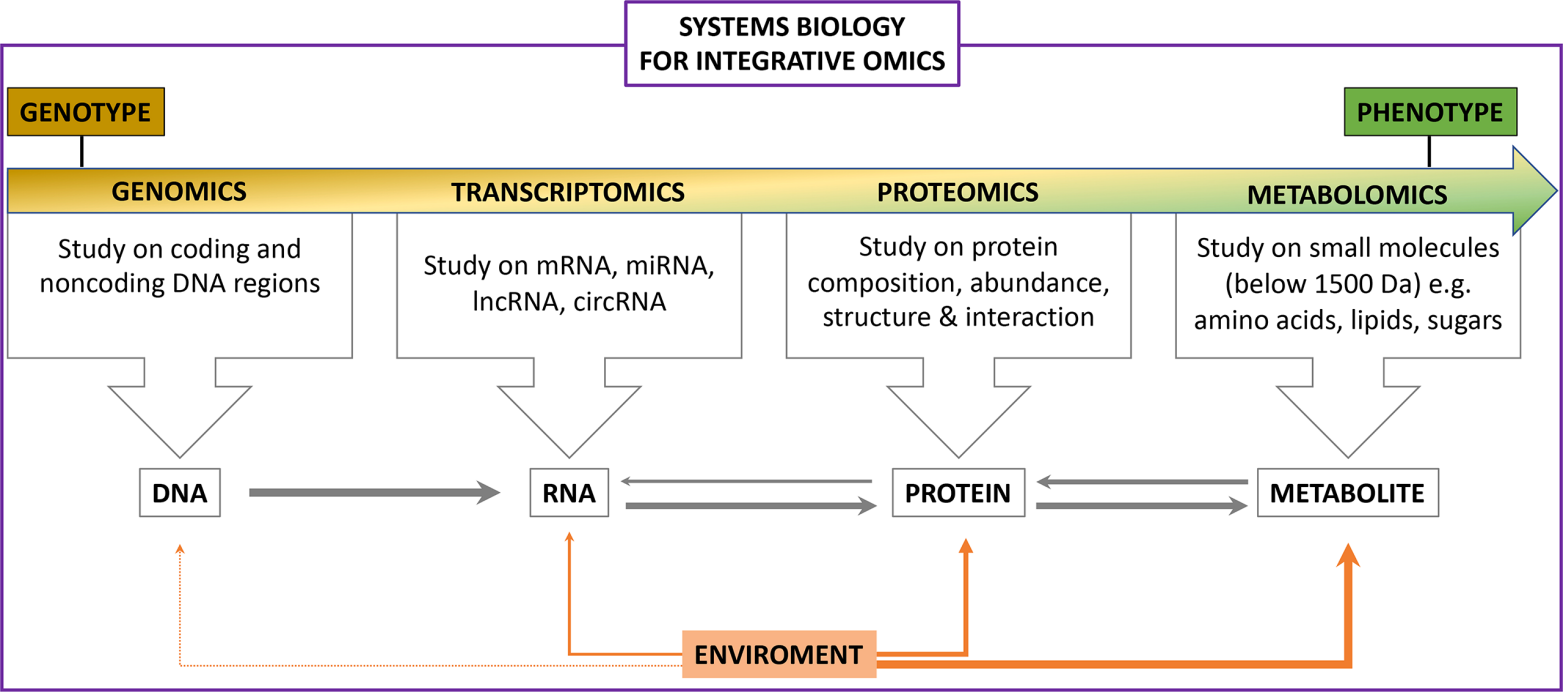
The scope of Integrative Omics.

Hu et al utilized whole-exome sequencing and transcriptome sequencing (RNA-seq) of

41 patients with parathyroid adenomas, confirming mutations in the adenomas and, additionally, identified the somatic mutation of EZH1. Subsequent RNA-seq data clustering analysis identified factors that could contribute to the parathyroid adenoma gene expression profile and may contribute to a clinically useful diagnostic panel if replicated in a larger cohort.

The value of an “omics” approach to therapeutic efficacy was undertaken by Martin et al in a model of diabetic kidney disease. Here, the authors looked at the medications that would activate tubular fatty acid oxidation following an animal model of bariatric surgery (Roux-en-Y) using transcriptomic and urinary metabolomic profiling. Medication-specific transcriptomic responses following Roux-en-Y surgery were explored using a network pharmacology approach. Their integrative multi-omic analyses suggested fenofibrate, as an agonist of peroxisome proliferator activated receptor-alpha (PPARa)-stimulated fatty acid oxidation, had potential utility in a combinatorial approach for the treatment of diabetic kidney disease in the setting of obesity. These interesting data need replication and clinical evaluation in human disease and may contribute to future progress in the treatment of diabetic kidney disease.

A further contribution to diabetic kidney disease was provided by Wang et al. This study was conducted in a human renal proximal epithelial tubular cell line with the aim to investigate molecular pathways contributing to glucose homeostasis under lactate dehydrogenase inhibition (oxamate), which was shown previously to modulate glucose metabolism by impacting mitochondrial respiration. The authors identified 3,884 genes there were significantly altered in the cell line under oxamate treatment and they linked those alterations with mitochondrial biogenesis and mitophagy which, when prolonged, compromises stress resistance. These data may indicate a molecular survival pathway in these cells, but replication in primary human tissue is required to indicate whether this may have potential clinical relevance.

Further insights into insulin resistance (IR) were provided by Yang et al. who utilized a proteomic approach to determine molecular dysregulations in white adipose tissue (WAT), liver and skeletal muscle of mice in which IR was induced with a high fat diet. A network-based approach was developed to explore IR-related tissue communications. Tissue specific insulin resistance networks were constructed with functional analysis of the cross-tissue interface. The results of this analysis suggested that the liver is a key player in insulin resistance as the liver was the only tissue expressing abnormal glucose metabolic signals. Moreover, the authors identified the CD36–PPAR axis in liver and WAT and verified it as a bridge that links cross-tissue signals with intracellular metabolism, potentially contributing to IR. Whilst there are limitations to this approach (animal model, static experiments, no other validation studies in the literature), the importance to humans may be in the relevance of the mechanisms of central IR and thus future molecular therapeutic approaches.

Diboun et al. utilized metabolomics in pregnant women in their second trimester with and without polycystic ovary syndrome (PCOS) and showed that women with PCOS had lower birthweight babies marked by a unique metabolic signature of specific triglycerides that, if confirmed in a larger population, may have diagnostic relevance in these patients.

Ding et al. also used metabolomics to investigate perimenopausal obesity compared to non-obese women in a Chinese population. In total, forty-six different metabolites were identified that may be useful as a metabolic signature to identify women at risk for perimenopausal obesity. Moreover, the authors linked those metabolites to dysfunction in dimethylarginine dimethylaminohydrolase (DDAH)/asymmetric NG, NGdimethyl−L−arginine (ADMA)/nitric oxide synthase (NOS)/nitric oxide (NO) signaling pathway related to the onset of cardiovascular disease. Nevertheless, replication studies in larger populations and in different ethnic backgrounds would be required to further translate this finding into clinical settings.

Continuing to focus on obesity, Joshi et al. used a bioinformatic approach to identify differentially expressed genes relevant for obesity that potentially could serve as therapeutic targets. The authors established databases from the available gene expression datasets and translated them into protein-protein and miRNA regulatory networks to identify key hub genes for obesity. Further, the authors identified, through bioinformatics analysis combined with validation genes, STAT3, CORO1C, SERPINH1, MVP, ITGB5, PCM1, SIRT1, EEF1G, PTEN and RPS2 as critical components in the development and prognosis of obesity; those genes could be probed as potential drug targets.

The importance of advanced bioinformatic approaches was also shown by Tsai et al. who deployed whole genome and chromatin immunoprecipitation sequencing data to provide further insight into osteoporosis. The authors investigated the association between RUNX2, a key molecule for osteoblast development, and functional SNPs to find factors contributing to osteopenia/osteoporosis and identified SNP rs6086746 (located upstream of the PLCB4 gene) as one of the factors involved in the etiology of osteopenia/osteoporosis. This study enhances the current understanding of the susceptibility to osteoporosis and further suggests the role of PLCB4 regulation in osteoporosis.

Overall, this series has highlighted that the “omic” approach yields powerful and extensive data for each of the areas explored and can provide further insight into disease etiology and disease progression as well as highlighting molecular targets for their treatment. However, there are significant challenges that must be addresses going forward. These include comparability of methodologies applied to describe each “omic”, strategies enabling integration of multi-omics, standardization of quality control, which will have to be applied to make the results reported readily comparable to one another and to clinical parameters. Moreover, there is a need to translate the findings into the clinical pipeline that will require investment of effort and resources into future clinical studies that implement multi-omic platforms. Thus, if we view human “omics” as a carpet with a complex interwoven pattern, then we appear to still be looking at the underside and thus are only seeing a few threads.

## Author Contributions

All authors listed have made a substantial, direct, and intellectual contribution to the work and approved it for publication.

## Conflict of Interest

The authors declare that the research was conducted in the absence of any commercial or financial relationships that could be construed as a potential conflict of interest.

## Publisher’s Note

All claims expressed in this article are solely those of the authors and do not necessarily represent those of their affiliated organizations, or those of the publisher, the editors and the reviewers. Any product that may be evaluated in this article, or claim that may be made by its manufacturer, is not guaranteed or endorsed by the publisher.

